# Opposite Effects of Early-Life Competition and Developmental Telomere Attrition on Cognitive Biases in Juvenile European Starlings

**DOI:** 10.1371/journal.pone.0132602

**Published:** 2015-07-29

**Authors:** Melissa Bateson, Michael Emmerson, Gökçe Ergün, Pat Monaghan, Daniel Nettle

**Affiliations:** 1 Centre for Behaviour & Evolution and Institute of Neuroscience, Newcastle University, Newcastle upon Tyne, United Kingdom; 2 Institute of Biodiversity, Animal Health & Comparative Medicine, University of Glasgow, Glasgow, United Kingdom; Università della Tuscia, ITALY

## Abstract

Moods are enduring affective states that we hypothesise should be affected by an individual’s developmental experience and its current somatic state. We tested whether early-life adversity, induced by manipulating brood size, subsequently altered juvenile European starlings’ (*Sturnus vulgaris*) decisions in a judgment bias task designed to provide a cognitive measure of mood. We predicted that starlings from larger broods, specifically those that had experienced more nest competitors larger than themselves would exhibit reduced expectation of reward, indicative of a ‘pessimistic’, depression-like mood. We used a go/no-go task, in which 30 starlings were trained to probe a grey card disc associated with a palatable mealworm hidden underneath and avoid a different shade of grey card disc associated with a noxious quinine-injected mealworm hidden underneath. Birds’ response latencies to the trained stimuli and also to novel, ambiguous stimuli intermediate between these were subsequently tested. Birds that had experienced greater competition in the nest were faster to probe trained stimuli, and it was therefore necessary to control statistically for this difference in subsequent analyses of the birds’ responses to the ambiguous stimuli. As predicted, birds with more, larger nest competitors showed relatively longer latencies to probe ambiguous stimuli, suggesting reduced expectation of reward and a ‘pessimistic’, depression-like mood. However, birds with greater developmental telomere attrition—a measure of cellular aging associated with increased morbidity and reduced life-expectancy that we argue could be used as a measure of somatic state—showed shorter latencies to probe ambiguous stimuli. This would usually be interpreted as evidence for a more positive or ‘optimistic’ affective state. Thus, increased competition in the nest and poor current somatic state appear to have opposite effects on cognitive biases. Our results lead us to question whether increased expectation of reward when presented with ambiguous stimuli always indicates a more positive affective state. We discuss the possibility that birds in poor current somatic state may adopt a ’hungry’ cognitive phenotype that could drive behaviour commonly interpreted as ‘optimism’ in food-rewarded cognitive bias tasks.

## Introduction

In humans, there is substantial epidemiological evidence that various forms of early-life adversity are associated with an increased probability of developing mood disorders such as anxiety and depression later in life [1–3]. However, rather less is currently known about the proximate and ultimate explanations for this relationship. Moods can be defined operationally as “relatively enduring states that arise when negative or positive experience in one context or time period alters the individual’s threshold for responding to potentially negative or positive events in subsequent contexts or time periods” [4]. The function of mood may be to integrate information about the recent state of the environment and the current physical condition of the individual in order to adaptively tune decisions about the allocation of behavioural effort [4–8].

Viewing moods as reflecting the adaptive adjustment of thresholds for responding to the possibility of positive and negative events potentially sheds some light on how and why early-life experience should affect adult moods. According to signal detection theory [9], the optimal threshold for responding to an ambiguous stimulus potentially signalling a meaningful event is influenced by two factors: the actual probability of the event occurring, and the relative costs of the two types of errors the individual can make, namely failing to respond to an event that occurs (a false negative decision) and incorrectly responding when there is no event (a false positive decision) [5]. It is a reasonable assumption that both of these factors could be influenced by an individual’s developmental experience, and moreover that such plasticity could be adaptive. The developing individual is exposed to a specific pattern of rewarding and punishing events and could use this information to estimate the probabilities of reward and punishment occurring in its future environment. Such a strategy would be adaptive if the features of environments are auto-correlated and change slowly relative to lifespan [10]. Moreover, the quality of the developmental environment is likely to influence the physical quality of the body an individual is able to develop, and this somatic state variable could influence the relative costs of response errors independent of the current environment in which an animal finds itself. For example, an animal that is less fast or less strong is more likely to succumb to predation if it fails to detect a predator and respond appropriately. Hence, all else being equal, predator detection thresholds should be adjusted to make false negative decisions less likely in animals in a poor somatic state [5]. Henceforth, we refer to this latter effect arising from the differential costs of response errors as an individual’s ‘vulnerability’ [5]. In general, we predict that it should be optimal for animals with higher punishment probabilities and/or higher vulnerabilities to set lower thresholds for responding to potential threats, with the consequence that they will exhibit an anxiety-like phenotype characterised by higher expectation of punishment in the face of ambiguous information [5]. Similarly, we predict that it should be optimal for animals with lower reward probabilities to set higher thresholds for responding to potential rewards, with the consequence that they will exhibit a depression-like phenotype characterised by lower expectations of reward in the face of ambiguous information [4]. Thus, we propose that the observed relationship between early-life adversity and subsequent negative moods could be the result of an evolved response that adapts the young animal to perform optimally given either the environment in which it expects to find itself and/or the constraints of the body it has been able to develop.

Here we use the European starling (*Sturnus vulgaris*) as an animal model in which to test experimentally the effects of manipulating biologically realistic aspects of developmental experience and resulting somatic state on subsequent mood. Starlings are a long-lived, non-domesticated passerine bird species commonly used in behavioural research [11,12], for which we have established manipulations of early-life adversity [13,14] and cognitive measures of mood [15–17]. For the current study, we had available a cohort of juvenile birds that had previously been subjected to a brood-size manipulation [18] in which pairs of wild focal siblings matched for weight were cross-fostered into nests where they faced either high (total of 7 chicks initially) or low (total of 2 chicks) competition for 12 days spanning the period during which most growth occurred (post-hatching day 3 to day 15, subsequently d3-d15). We have previously shown that this manipulation caused differences in early growth, with the chicks from high-competition broods being on average smaller than chicks from low-competition broods during the period of the manipulation. This difference occurred because within the high-competition nests, some chicks lost out in the increased competition for food [18]. Although these differences in weight rapidly disappeared once the chicks were released from the manipulation onto *ad libitum* food, there were enduring differences in the length of telomeres measured from the birds’ erythrocytes. Specifically, telomere attrition over the developmental period was predicted by the number of competitors above the focal chick in the brood hierarchy of size (i.e. heavier than the focal chick) on d15: chicks with more heavier competitors on d15 experienced greater developmental telomere attrition and this difference was still evident at d55 [18]. This result is relevant to the current study because telomere length in humans and birds measured from blood prospectively predicts health and life-expectancy [19–24], and is therefore emerging as a plausible candidate measure of somatic ‘state’ as defined by behavioural ecologists [25]. On the basis of these results, it is likely that the number of heavier competitors in the nestling phase affected the developing birds’ experience of the probabilities of reward and punishment, as well as producing enduring changes in their somatic state that affected their vulnerability. Our aim was to investigate whether the birds differed in mood as predicted by the theory described above.

We measured mood using a cognitive bias task [26,27]. Such tasks are designed to assess response thresholds in the face of ambiguous information about potential punishment or reward, and are hence ideally suited for testing the theoretical framework for mood outlined above [4]. The literature exploring the use of cognitive biases to assess mood in non-human animals has focused on tasks that measure judgment biases in which animals are asked to interpret ambiguous information (for reviews of empirical papers using the cognitive bias approach see [27–30]). In a typical judgment bias task, such as that designed by Bateson and Matheson for starlings [15], subjects are initially trained to associate one stimulus—positive—with a high-valued reward and another stimulus—negative—with either punishment or lack of reward. Once the subjects have acquired the discrimination between the positive and negative stimuli, they are subsequently tested by presenting them with ambiguous stimuli intermediate between the two trained stimuli. In the test trials, animals that respond to the ambiguous stimuli similarly to the positive stimulus are interpreted as displaying a high expectation of reward in the presence of ambiguous information, and hence an ‘optimistic’ cognitive style indicative of a positive mood. In contrast, animals that respond to the ambiguous stimuli similarly to the negative stimulus are interpreted as displaying a higher expectation of punishment or lower expectation of reward, and hence a more ‘pessimistic’ cognitive style indicative of a more negative mood. Such cognitive bias tasks are currently regarded as the gold standard for assessing moods in non-human animals because they test clear, *a priori* predictions about the relationship between behavioural decisions and mood that emerge from a general operational definition of mood [4,8,27]. Although cognitive bias tasks have been used to assess the effects of various acute and chronic manipulations of adult animals in a variety of species, they have not thus far been extensively used to explore lasting effects of past developmental experience (for a couple of exceptions in rats see [31,32]).

The aim of the current experiment was to ask whether our brood-size manipulation in nestling starlings led to differences in cognitive biases in the juvenile birds indicative of altered mood. We hypothesised that differential competition early in development would have altered the probabilities of both reward and punishment experienced by the birds. Chicks at the bottom of the hierarchy in high-competition nests received less food (lower reward probabilities) and this was plausibly coupled with greater stress from competition with nest mates (higher punishment probabilities). We also hypothesised that shorter telomeres in the juvenile birds would be indicative of poorer somatic state, and hence increased vulnerability. Together these hypotheses suggest that birds from the bottom of the hierarchy in high-competition nests and birds with shorter telomeres (note that these are not always the same birds, because the relationship between number of heavier competitors and telomere attrition is not perfect) should have reduced expectations of reward and increased expectations of punishment in the face of ambiguous information, indicative of depression-like and anxiety-like moods respectively. If these assumptions are correct, then we predict that birds from high-competition nests should display more pessimistic decisions than birds from low-competition nests when tested on a cognitive bias task. Furthermore, we expect the relationship to be stronger when we use number of heavier competitors as the predictor variable, since this seems to capture more of the variation in relevant developmental experience than brood size.

## Methods

### Ethics statement

Our study adhered to the ASAB/ABS Guidelines for the Use of Animals in Research, and was approved by the local ethical review committee at Newcastle University. It was completed under UK Home Office project licence number PPL 60/4073, and removal of starlings from the wild was authorised by Natural England (licence number 20121066). After the completion of the current experiment the birds were retained in the laboratory for further studies before being permanently rehomed to a large outdoor aviary in July 2013. To maximize welfare during experiments, birds were tested in home cages rather than being repeatedly caught, as described below. Birds were tested for 4 hours per day and had *ad libitum* food and baths at other times. Between experiments, all birds lived in social groups in large free-flight aviaries enriched with foraging substrate, multiple perches, and baths.

### Subjects

Subjects were 31 European starlings (15 males and 16 females) from a cohort of chicks hatched in the wild (in nest boxes located on five farms in Northumberland, UK: approximately 55˚N 2˚W) in May 2012. Whilst still in the wild, the chicks were subjected to a brood size manipulation described in detail elsewhere [18] but summarised briefly below.

Using our nest box colonies of starlings, we identified sets of nests in which chicks hatched on the same day. We weighed all chicks on the day after hatching (d2), and selected donor nests containing at least four chicks of approximately the same weight. Within each donor nest, the four chicks with the most similar weights became our four focal chicks. On d3, we moved a randomly chosen pair of each set of focal chicks to a host nest where they would be the only nestlings (brood size of two: the low competition treatment), whilst the remaining pair were moved to a different host nest in which we additionally placed five competitors (brood size of seven: the high competition treatment). Nests of seven chicks are within the observed range of natural variation in this population of starlings, and this manipulation has been used previously without causing chick mortality [14]. The additional competitors in the broods of seven were not siblings of the focals, and also were not in their natal nests. In this way, we created nine sets of four focal siblings (36 birds), but one low competition brood was abandoned on d4 and the chicks died leaving 34 focal birds. In high competition broods, where a non-focal competitor died within the first three days post-manipulation, we replaced the dead individual with another chick of approximately the same weight. Due to mortality at later stages, one of the high competition broods contained only six live chicks on d15, and one nest 5. We weighed the focal chicks on d4, d7, d11 and d15. In addition, on d15, we weighed all of the non-focal competitor chicks from the large broods. The brood size manipulation created continuous variation in the number of larger nest competitors chicks faced on d15 (0 to 6).

On d15, the 34 surviving focal chicks were taken from their nests and reared in captivity. Once in captivity, birds from the two treatments were mixed together, and housed in covered buckets until fledging at around d21. Before fledging birds were fed to satiation on commercial poultry-based cat foods mixed with apple sauce and added vitamins and minerals (full details are provided in Feenders and Bateson [33]. After fledging, birds were group-housed, initially in cages and later (around d30 onwards) in large indoor aviaries (215 × 340 × 220 cm WDH; ~18°C; 40% humidity; 13L:11D light cycle). The birds were provided with clean drinking water and fed *ad libitum* on commercial poultry-based cat foods, fruit, commercial grain-based chick starter crumbs, live mealworms (*Tenebrio molitor*) and dried insect pate (Orlux). They were additionally provided with environmental enrichment in the form of water baths, wood chips for probing and ropes and hanging cardboard boxes for perching and roosting. Birds were weighed in captivity at d20 and d55 (± 2 days). Tarsus length was measured at d55. One bird died after fledging but before d55. Of the remaining 33 birds 31 served as subjects in the current study; the two birds not used were the pair for which we did not have a pair of siblings from the low competition treatment.

Measurements of cognitive bias took place when the birds were independent fledglings starting on a mean of d94 post-hatch. Three replicates of 8 birds and one replicate of 7 (each comprising 2 genetic families) were sequentially caught from the aviary and moved to our experimental laboratory (~18°C; 40% humidity; 13L:11D). Training started on d68 (± 2 days) for replicate 1, d80 (± 2 days) for replicate 2, d96 (± 2 days) for replicate 3 and d119 (± 2 days) for replicate 4. Birds were housed in individual cages that served both for testing and as their home cages for the duration of the cognitive bias experiment. The cages measured 100 x 45 x 45 cm (WDH) and were furnished with two perches and a water bath. Water was always available *ad libitum*. Food was available *ad libitum* other than during experimental sessions, which ran approximately 0800–1200 daily, when food bowls were removed from the cages. Birds were in auditory contact with each other throughout the period in cages and were visually isolated using curtains for the period of the daily experimental sessions. Each replicate remained in the cages for approximately 2 weeks after which the birds were returned to the aviary and were replaced with the next two families.

Birds were weighed when they were caught for transfer to individual cages at the start of the experiment. As a measure of body condition at the start of the experiment we derived residual body weights from the regression equation: Weight = 1.717*Tarsus + 15.437 obtained from all 31 birds on d55.

### Cognitive bias task

#### Task overview

The task was based on the go/no-go judgment bias task developed by Bateson and Matheson [15] in which individual starlings were presented with a single Petri dish containing a mealworm covered with a circular card lid. The bird was required to make a decision about whether to approach and remove (i.e. probe) the lid during a time-limited presentation of the dish. The colour of the lid provided a discriminative stimulus indicating the palatability of the hidden worm. Birds were initially trained to discriminate two shades of grey lid, one associated with a palatable worm (POS), and the other associated with a noxious worm (NEG), and were subsequently tested with ambiguous lids intermediate between the two trained achromatic colours, presented in extinction. The achromatic colours used were 20%, 30%, 40%, 50% and 60% grey, printed in black on white card. Each bird was assigned one extreme (i.e. 20% or 60%) as the POS stimulus and the other as the NEG stimulus; colour assignments were the same within a replicate, but counterbalanced across replicates, so two replicates had 20% grey as POS and two replicates had 60% grey as POS. The three intermediate grey shades were used as the ambiguous stimuli in the test trials. Palatable mealworms were injected with 0.02ml water and painted externally with water, whereas noxious mealworms were injected with 0.02ml of 4% quinine sulphate solution and painted externally with the same solution. The task comprised four phases: lid-flipping training, discrimination training, partial reinforcement training and cognitive bias testing.

#### Lid-flipping training

The aim of this phase was to train the birds to approach and remove a cardboard lid from a Petri dish to access a palatable mealworm hidden beneath. Birds were food deprived for one hour prior to the start of training to increase their motivation to approach the dish. Training started with the POS lid placed adjacent to a dish containing two mealworms; the dish was presented for 10 min during which the experimenter left the room. In the course of subsequent training the number of worms was reduced to one, the lid was placed so that it gradually covered progressively more of the dish, the presentation time was reduced to 1 min and the experimenter stayed in the room hidden behind a curtain. In the final stage, birds were given sessions of 8 sequential trials separated by an inter-trial interval (ITI) of 5 min in which the lid fully covered the worm and the dish was presented for 1 min. Birds were required to flip the lid and eat the worm on 6 out of 8 trials before they were allowed to progress to discrimination training.

#### Discrimination training

The aim of this phase was to train the birds that one shade of lid, the positive stimulus (POS), was associated with a palatable mealworm and another shade of lid, the negative stimulus (NEG), was associated with a noxious mealworm. Birds should probe POS lids and refrain from probing (or be slower to probe) NEG lids. No food deprivation was necessary in this or subsequent phases of the experiment; indeed birds were pre-fed with 12 mealworms in the 30 mins prior to the experimental sessions in an attempt to reduce their motivation to probe lids. Birds received one session of 16 trials per day with an ITI of 5 minutes. In all trials the lid completely covered a mealworm and the dish was presented for 1 min before being removed from the cage. In 8 trials the POS lid covered a palatable mealworm as in lid-flipping training. In the other 8 trials the NEG lid covered a noxious mealworm. Trials were presented in a pseudorandom order with the constraint that all sessions started with a POS trial and no more than two trials of the same type occurred sequentially. To assess whether birds had learnt the discrimination, each day we compared the individual birds’ latencies to probe in POS and NEG trials. The emergence of significant discrimination during discrimination training was established by daily comparing each bird’s latencies to probe POS and NEG lids using Mann-Whitney U tests. Non-parametric tests were chosen for these analyses due to very small sample sizes. The criterion for allowing a bird to progress to partial reinforcement was that it was significantly faster to probe POS lids than NEG lids in two sessions.

#### Partial reinforcement training

The aim of this phase was to train the birds that not all trials were reinforced or punished in order to slow down extinction of lid flipping in the subsequent cognitive bias tests. Birds received one session of 16 trials comprising: 4 POS trials reinforced with a palatable worm (exactly as in discrimination), 4 POS trials with no worm, 4 NEG trials punished with a noxious worm (as in discrimination) and 4 NEG trials with no worm. The ITI was 5 mins. The trials were presented in a pseudorandom order with the constraint that the session started with a reinforced POS trial and no more than two POS or NEG trials occurred sequentially.

#### Cognitive bias testing

The aim of this phase was to measure the birds’ response to novel, ambiguous stimuli (Near POS, MID and Near NEG) intermediate between the two trained stimuli (POS and NEG). Birds received four daily sessions of 18 trials, each session comprising: 4 POS trials reinforced with a palatable worm, 2 POS trials with no worm, 2 Near POS trials with no worm, 2 MID trials with no worm, 2 Near NEG trials with no worm, 2 NEG trials with no worm and 4 NEG trials punished with a noxious worm. The ITI was 5 minutes. The trials were presented in a pseudorandom order with the constraint that the session started with a reinforced POS trial and no more than two trials of the same type occurred sequentially. The maintenance of significant discrimination of POS and NEG during judgment bias testing was checked by comparing each bird’s latencies to probe POS and NEG lids over the four days of testing using Mann-Whitney U tests.

#### Data collection

Data were recorded by an observer (MB, ME, GC or DN) in an adjacent room watching a live video showing an aerial view of the 8 bird cages (videos of all sessions were also recorded for subsequent inter-observer reliability checking). During data collection the experimenters and observers were blind to the brood-size treatment group to which each bird belonged (birds were individually identified by the combination of coloured leg rings they wore) to prevent experimenter bias. In all phases of the experiment we used a stopwatch to record the latency of the bird to probe the lid (defined as touching it with its beak) and to eat the mealworm if one was present in that trial. The latency began when the experimenter’s hands exited the cage. If the bird did not probe the lid (a no-go response) or did not eat the mealworm within the specified time limit, the latency was scored as the maximum plus 1 s (i.e. 61 s in discrimination training and judgment bias testing). Since many birds appeared unable to inhibit probing NEG cues, even after several days of training (see results), we used latency to probe, rather than whether a probe took place or not, as our dependent variable in the cognitive bias test.

### Developmental telomere attrition

Telomere lengths on d4, d15 and d55 were measured from erythrocyte DNA using quantitative PCR and have been published previously [18]. Whilst the estimates of telomere length derived from quantitative PCR are not absolute and can be affected by the presence of interstitial repeats of the telomeric sequence, neither of these criticisms applies if longitudinal measurements from the same individual are compared, as was done in the current study [34]. Due to some failed assays, telomere length data were only available for 22 of the 30 birds included in the current study (of these 22 birds, 11 came from each brood size treatment, meaning that treatment did not bias the failure of the telomere assays). As a measure of developmental telomere attrition we calculated *D*, the difference in telomere length between d4 and d55 corrected for regression to the mean using the equation given in Verhulst et al. [35]. More negative values of *D* correspond to greater telomere attrition. Since *D* remained weakly negatively correlated with telomere length at d4 (Pearson correlation: r(20) = -0.16), we included telomere length at d4 as a covariate in all analysis of the effects of *D* [36].

### Data Analysis

Raw data from the study are available in S1, S2 and S3 Files. Data were analysed using R [37]. The R script is available on request. A criterion for significance of p < 0.05 was assumed throughout; results with p < 0.10 are also reported and discussed as marginally non-significant trends.

For principal component analysis (PCA) we used the R package ‘psych’ [38]. To model the data we used general linear mixed models (GLMMs) implemented in the R package ‘nlme’ [39]. Model estimation was by maximum likelihood, and whether parameters differed significantly from zero was determined by testing the change in deviance when a given predictor was excluded from the model using a X^2^ test.

All GLMMs included random intercepts for genetic family (since quartets of birds were siblings) and also bird when repeated measures were present; when both random effects were necessary, bird was nested in genetic family. Predictor variables explored in the models included: developmental treatment (categorical: broods of 2 or 7 chicks), number of heavier competitors that a chick had in the nest at d15 (continuous: 0–6; see Table 1), telomere attrition between d4 and d55 (*D*; continuous, negative values indicate more attrition), current weight at the start of the cognitive bias experiment (continuous), current body condition at the start of the cognitive bias experiment (continuous; larger values indicate birds that were heavier for their size), stimulus valence in the cognitive bias experiment (continuous: 1–5, where 1 = NEG and 5 = POS). Some models additionally controlled for sex (categorical: male or female), telomere length at d4 (continuous) and average speed to probe POS and NEG (continuous; see below). We experimented with adding experimental replicate (1–4) to the models as a predictor, but it did not improve model fit, and we therefore elected to eliminate it from the final models reported in the interests of simplicity. The fixed effects included in each model are detailed in the results section.

**Table 1:** Distribution of focal chicks with different numbers of heavier competitors at d15 in the two brood size treatments and in total.

Number of heavier competitors at d15	Number of focal chicks		
	Low-competition treatment (brood size 2)	High-competition treatment (brood size 7)	Total
**0**	7 (4)	3 (2)	10 (6)
**1**	8 (7)	4 (3)	12 (10)
**2**	0	1 (1)	1 (1)
**3**	0	1 (1)	1 (1)
**4**	0	0	0
**5**	0	2 (1)	2 (1)
**6**	0	4 (3)	4 (3)
**Total**	15	15	30 (22)

Note: the numbers in brackets give the number of birds in the subset of the data for which telomere length measures were available.

For the analysis of the cognitive bias data we used the behaviour of birds in individual trials as the unit of analysis. Our main dependent variable, latency to probe a lid, was log_e_-transformed prior to GLMM analysis to reduce the effect of outliers. Although latencies to probe in the cognitive bias test trials were theoretically bounded between 0 and 61 s, inspection of residuals from the fitted models showed that assumptions of normality and homogeneity of variance were not violated, hence a Gaussian error structure was assumed throughout. No adjustment was made for censoring since birds responded on or before the limit of 60 s in 78% of the ambiguous test trials.

For the main analysis of the effects of treatment on latency to probe, we first report a model using the data from the trained POS and NEG trials only (valences 1 and 5). The purpose of this model is to establish whether developmental treatment predicts differences in the birds’ overall speeds of probing. We subsequently report models using the data from the ambiguous trials only (valences 2,3 and 4), but including the birds’ average speed (latency to probe POS and NEG) as a covariate. The purpose of these models is to establish whether our predictor variables predict different latencies to probe the ambiguous stimuli in particular, once variation in birds’ overall speed is accounted for (for a similar approach to the analysis of cognitive bias data see [40]). Alternative models of the data were compared using AIC_c_, a modified version of Akaike’s information criterion recommended for small sample sizes [41].

## Results

### Measures of early-life adversity and current state

Although the birds used in the current study originated from a designed experiment in which sibling chicks were allocated to broods of either 2 (low-competition treatment) or 7 (high-competition treatment) chicks, previous analyses of the effects of treatment on chick weight gain showed that whilst there were overall effects of treatment, these were being driven by loser chicks in the high-competition treatment [18]. For this reason, we have argued previously that continuous measures, such as either the number of heavier competitors that a chick had on d15 [18,25] or its weight on d11 [42], are more precise indices of early-life adversity than simply whether a chick was in the high- or low-competition treatment.

In order to clarify which predictor variables to include in subsequent models we first explored how the brood size treatment affected various putative continuous measures of early-life adversity and current state. Using the data from the 30 birds for which we obtained behavioural measures in the current experiment, we conducted a series of GLMMs to test the effect of treatment on the following variables: weight at d11 (the day on which the difference in growth of chicks from the two treatments was largest [18]), number of heavier competitors at d15, developmental telomere attrition between d4 and d55, current weight at the start of the cognitive bias experiment and current body condition at the start of the cognitive bias experiment. In models of variables related to weight we included sex as an additional predictor because in European starlings males are on average heavier than females. In the model of developmental telomere attrition we included telomere length at d4 in the model because we had previously established that this explains some of the variation. The results are shown in Table 2. Treatment had a significant effect on weight at d11 and the number of heavier competitors that a chick had at d15, with chicks from the high competition treatment (broods of 7) being lighter on d11 and having more heavier competitors on d15. Note that the latter result is a direct consequence of our manipulation, because in low-competition broods (2 chicks) a chick could only have either 0 or 1 heavier competitors, whereas in high-competition broods (7 chicks) a chick could have up to 6 heavier competitors. Although birds from high-competition broods experienced greater telomere attrition between d4 and d55 than birds from low competition broods, the effect of treatment was not significant, repeating our previously published findings from the same cohort of birds [18]. There was no lasting effect of the brood size treatment on either current body weight or current body condition.

**Table 2:** Summary of models testing the effect of the brood size treatment on other potential continuous measures of early-life adversity and current state.

Dependent variable	Predictor(s)	B ± se	Χ^2^	P-value
**Log**_**e**_ **(No. heavier competitors+1 on d15)**	Treatment (7)	0.74 ± 0.22	10.30	0.001*
**Weight d11**	Treatment (7)	-9.38 ± 2.07	15.99	< 0.001*
	Sex (F)	-5.77 ± 2.11	7.00	0.008*
**Telomere attrition d4-d55**	Treatment (7)	-0.23 ± 0.20	1.38	0.241
	Telomere length on d4	-0.13 ± 0.14	0.86	0.353
**Current weight ~d94** ^**§**^	Treatment (7)	0.58 ± 1.27	0.23	0.634
	Sex (F)	-5.24 ± 1.31	13.59	< 0.001*
**Current condition ~ d94** ^**§**^	Treatment (7)	1.42 ± 1.14	1.67	0.196
	Sex (F)	-3.49 ± 1.18	8.28	0.004*

Notes:

^§^Current weight/condition was measured on d68 for replicate 1, d80 for replicate 2, d96 for replicate 3 and d119 for replicate 4.

* P < 0.05. All models contain a random effect for genetic family. All models are based on the 30 birds for which we collected cognitive bias data with the exception of the telomere attrition model, that is based on the subset of 22 birds for which we additionally had telomere length data. Where predictor variables are categorical, parameter estimates for Treatment are for brood size 7 and for Sex are for females.

To explore how the above putative measures of early-life adversity and current state were related to one another, we constructed a correlation matrix. The results are shown in Table 3. Birds with more heavier competitors on d15 were lighter on d11, had greater developmental telomere attrition between d4-d55 and were also lighter at the start of the cognitive bias experiment (~d94). Birds that were lighter at the start of the cognitive bias experiment also had lower current body condition (because there was little variation in adult tarsus length). However, current body condition was not correlated with any measures of early-life adversity (neither number of heavier competitors, nor weight at d11 nor developmental telomere attrition).

**Table 3:** Correlation matrix showing how potential measures of early-life adversity and current state relate to one another.

	No. heavier competitors d15	Weight d11	Telomere attrition d4-d55	Current weight ~d94
**Weight d11**	r = -0.7			
	n = 30			
	P < 0.001*			
**Telomere attrition d4-d55**	r = -0.47	r = 0.32		
	n = 22	n = 22		
	P = 0.028*	P = 0.146		
**Current weight ~d94**	r = -0.39	r = 0.38	r = 0.01	
	n = 30	n = 30	n = 22	
	P = 0.034*	P = 0.039*	P = 0.970	
**Current condition ~d94**	r = -0.19	r = 0.06	r = -0.15	r = 0.90
	n = 30	n = 30	n = 22	n = 30
	P = 0.328	P = 0.755	P = 0.506	P < 0.001*

Notes. Each cell contains: the Pearson product-moment correlation coefficient (r), the number of birds on which the test is based (n) and the P-value for the correlation (P).

*P < 0.05.

### Discrimination training

All birds initially successfully learned lid-flipping, though one subsequently stopped reliably lid-flipping and never reached the criterion for progression to discrimination training. The following analyses are based on the 30 birds that successfully completed all phases of training, or where telomere attrition is a predictor, the subset of 22 birds for which telomere attrition data was available. Birds took 10.63 ± 5.13 (mean ± sd) trials to learn the lid-flipping task, where success was defined as the bird removing a lid fully covering a mealworm within 60 s of the start of the trial. To test whether brood size treatment predicted speed of learning we fitted a model with number of trials to learn lid flipping as the dependent variable and treatment as a fixed predictor. There was no effect of treatment on the number of trials taken to acquire lid flipping (GLMM: B for high-competition treatment ± se = 0.23 ± 1.84, X^2^(1) < 0.02, p = 0.896). We also found no effects of either number of heavier competitors on d15 or developmental telomere attrition (statistics not shown).

In contrast to the previous occasion on which we used this task [15], most birds continued to probe the NEG lids and some continued to eat the noxious mealworms after experiencing them multiple times. In the final 2 sessions of discrimination training the proportions of POS and NEG lids probed were 0.96 ± 0.18 (mean ± sd) and 0.51 ± 0.30 respectively, and the proportions of worms eaten were 0.95 ± 0.18 and 0.19 ± 0.23 respectively. Based on their latencies to probe, all 30 remaining birds successfully acquired the discrimination task. Birds took 2.03 ± 0.85 (mean ± sd) sessions to show a significant difference in their latency to probe POS and NEG lids with a shorter latency to probe POS lids (Mann-Whitney tests, all p < 0.050). To test whether there was an effect of treatment on discrimination learning we fitted a model with number of sessions to acquire the discrimination as the dependent variable and treatment as a fixed predictor. There was no significant effect of treatment on the number of sessions taken to learn the discrimination (GLMM: B for high-competition treatment ± se = 0.37 ± 0.24, Χ^2^(1) = 2.34, p = 0.126). We also found no effects of either number of heavier competitors on d15 or developmental telomere attrition (statistics not shown). Thus, there was no evidence that the developmental experience of the birds affected their ability to learn either lid flipping or an arbitrary, achromatic colour discrimination.

All 30 birds retained their discrimination between the POS and NEG stimuli during the four days of cognitive bias testing. Comparing the latencies from all of the POS and NEG trials (i.e. pooling reinforced and extinction trials) from the 4 days of testing (a total of 24 POS and 24 NEG for each bird), all birds remained significantly faster to probe POS than NEG stimuli (Mann-Whitney tests, p < 0.050). Therefore, all 30 birds were retained in the analysis of the cognitive bias data.

### Cognitive bias testing

#### Effects of brood size treatment on cognitive bias

**Fig 1:**
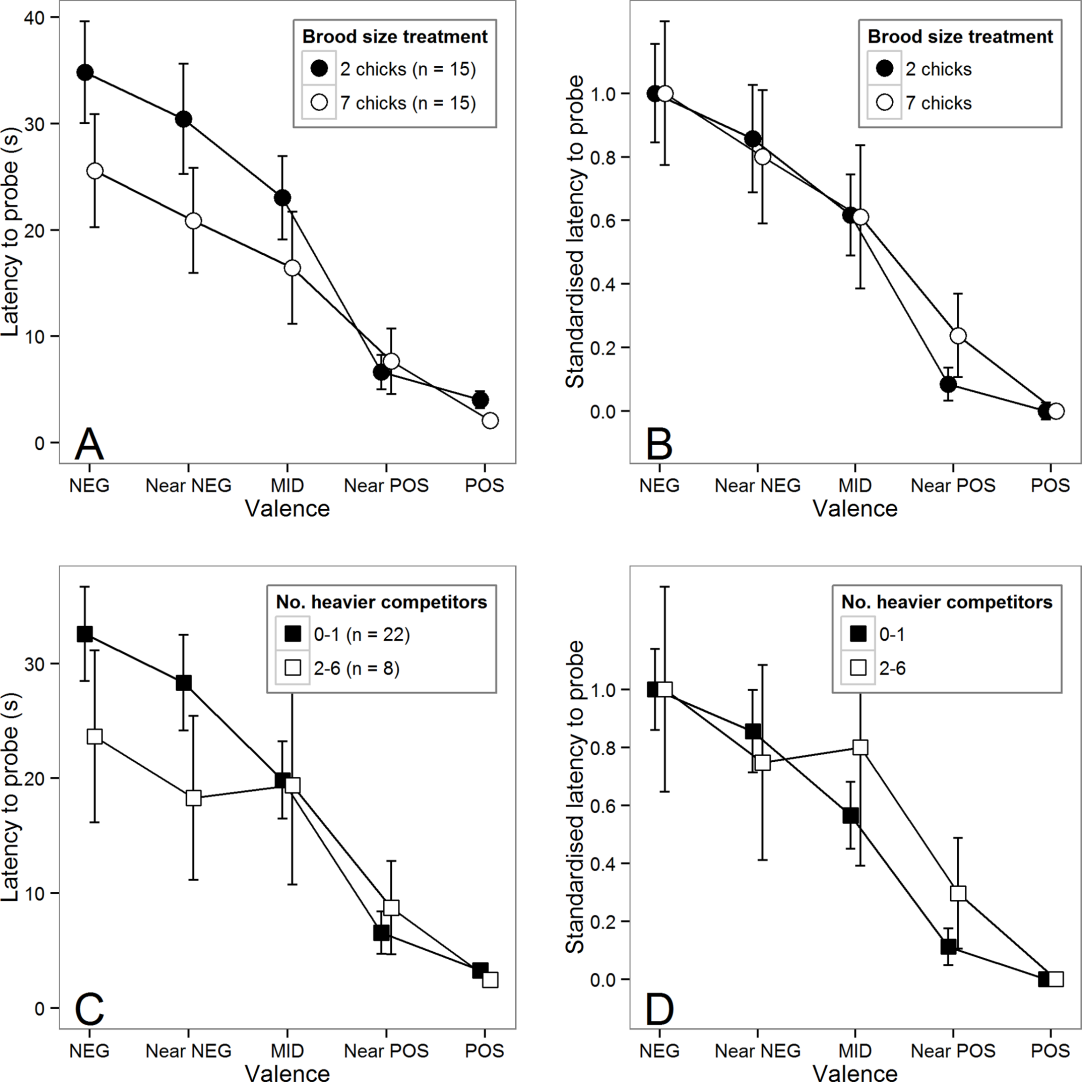
Effects of developmental competition on judgment bias. (A) Latency to probe as a function of stimulus valence for birds in the two brood-size treatments. Data are mean ± 1 s.e. latency to probe in the cognitive bias test trials. (B) The same data shown in panel A standardised so that the latencies to probe POS and NEG are 0 and 1 respectively. This standardisation removes differences in speed to probe POS and NEG between treatments, and hence reveals the differences in the shapes of the generalisation gradients between treatments. Note that this standardisation is for visualisation purposes only and was not used in the data analysis (see text for details). (C) Latency to probe as a function of stimulus valence for birds at the top of the weight hierarchy in the nest (0 or 1 heavier competitors) and birds at the bottom of the weight hierarchy (2–6 heavier competitors). Data are mean ± 1 se latency to probe in the cognitive bias test trials. The dichotomization of the data into the groups 0–1 and 2–6, is for visualisation purposes only; all statistical analyses were conducted using the number of heavier competitors as a continuous predictor variable. (D) The same data shown in panel C standardised so that the latencies to probe POS and NEG are 0 and 1 respectively. Note that the standard errors shown on all of the plots in this figure give a false impression (underestimate) of the significant differences between the groups due to the fact that birds from the same genetic family are present in both treatment groups.

Fig 1A shows that birds from the high-competition treatment were faster to probe all but the Near POS stimulus. Our first step was to explore whether there was an effect of treatment on latency to probe just the trained stimuli (POS and NEG). We fitted a model with latency to probe POS and NEG stimuli (logged) as the dependent variable and stimulus valence (1 or 5), brood size treatment and their interaction as fixed predictors. Valence significantly predicted latency to probe, with birds probing POS stimuli faster (GLMM: B for POS±s.e. = -2.07±0.07, X^2^(1) = 1003.91, p < 0.001). Brood-size treatment had a significant main effect on latency to probe, with birds from high-competition nests probing faster (GLMM: B for high-competition nests±s.e. = -0.68±0.26, X^2^(1) = 3.97, p = 0.046). There was a significant interaction between valence and treatment reflecting a greater effect of treatment on the latency to probe NEG stimuli (GLMM: B±s.e. = 0.29±0.10, X^2^(1) = 8.45, p = 0.004). Since this analysis shows an effect of treatment on the latency of the birds to probe the trained stimuli, we calculated the mean probe latency to the POS and NEG stimuli for each bird (henceforth its ‘speed’), and used this as a covariate in the following analysis of latencies to probe the ambiguous stimuli (Near POS, MID and Near NEG).

To explore whether treatment affected the birds’ response to the ambiguous stimuli in particular, we fitted a model with latency to probe ambiguous stimuli (logged) as the dependent variable and the following fixed predictors: speed (continuous), valence (2–4), brood-size treatment and the valence by treatment interaction. There was a significant main effect of valence on latency to probe the ambiguous stimuli, with birds being faster to probe stimuli more similar to POS (GLMM: B±s.e. = -0.73±0.07, X^2^(1) = 140.53, p < 0.001). The main effect of treatment was not significant, but there was a marginally non-significant interaction between valence and treatment on latency to probe, that occurred because birds from low-competition nests were faster to probe Near POS cues than birds from high-competition nests (GLMM: B = 0.20 ± 0.10, X^2^(1) = 3.79, p = 0.052; Fig 1B).Thus, when overall speed is controlled for, as predicted, there is some evidence, albeit marginally non-significant, that birds from small broods had a higher expectation of reward in the face of ambiguous information.

#### Effects of number of heavier competitors on cognitive bias

In an attempt to find a better model of the cognitive bias data we explored replacing the dichotomous treatment variable with the related, but potentially more precise, continuous measure of early-life adversity: ‘number of heavier competitors at d15’. Fig 1C and 1D show the same data plotted in Fig 1A and 1B but split by the number of heavier competitors at d15 (the continuous variable was divided into two groups of 0–1 and 2–6 for presentation purposes only). Fig 1C shows that birds that had more, heavier competitors were faster to probe stimuli at the NEG and Near NEG end of the continuum.

Following the same analysis strategy adopted for the effects of treatment above, we fitted a model with latency to probe the ambiguous stimuli (logged) as the dependent variable and the following fixed predictors: speed, valence (2–4), number of heavier competitors, and the valence by number of heavier competitors interaction. There were significant effects of speed and valence on latency to probe the ambiguous cues (GLMM: for speed B ± se = 0.08 ± 0.01, X^2^(1) = 45.50, p < 0.001, and for valence B ± se = -0.75 ± 0.07, X^2^(1) = 140.53, p < 0.001). The main effect of number of heavier competitors was not significant (GLMM: B ± se = -0.16 ± 0.07, X^2^(1) = 0.21 p = 0.644), but there was now a significant interaction between valence and number of heavier competitors on latency to probe, that occurred because birds with fewer heavier competitors were faster to probe both MID and Near POS cues than birds with more heavier competitors (GLMM: B = 0.07 ± 0.02, X^2^(1) = 8.14, p = 0.004; Fig 1D). The fit of this latter model was better than the previous model presented above containing treatment as a predictor in place of number of heavier competitors (reduction in AIC_c_ of 4.261). Thus, the patterns are similar with treatment and number of heavier competitors as predictors, but number of heavier competitors captures more of the variance in birds’ latencies to probe ambiguous stimuli. As predicted, birds from broods where they faced fewer heavier competitors had a higher expectation of reward in the face of ambiguous information.

#### Effects of developmental telomere attrition on cognitive bias

**Fig 2:**
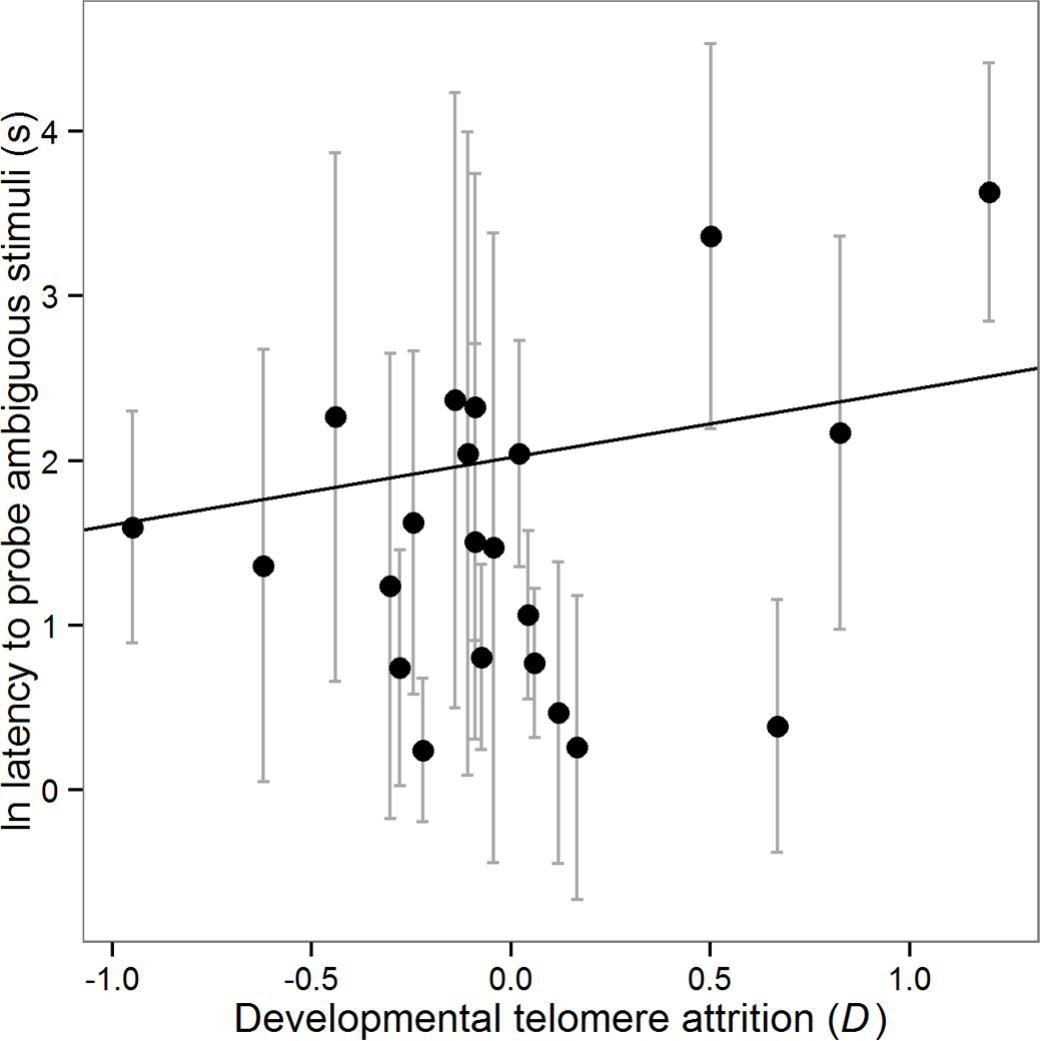
Effects of developmental telomere attrition on judgment bias. Latency to probe as a function of telomere attrition score. Telomere attrition score is the difference between telomere length at d4 and d55 adjusted for regression to the mean (see methods for details); negative values correspond to greater telomere loss. Data points are the mean ± 1s.d. of the ln latency to probe in the 24 judgment bias test trials with ambiguous stimuli (i.e. valences 2–4) for each bird (n = 22). The solid line shows the predicted regression line derived from the model described in the main text.

Since we have previously shown that number of heavier competitors at d15 predicts developmental telomere attrition in the same group of birds, we next explored whether developmental telomere attrition statistically mediates [43] the relationship between number of heavier competitors and cognitive bias established above. Developmental telomere attrition was correlated with number of heavier competitors, but not so strongly as to preclude entering both predictors into the same model and their having separate effects (r = -0.47, see Table 3). As previously, the dependent variable was latency to probe the ambiguous stimuli (logged). The fixed predictors initially included were: speed, valence (2–4), heavier competitors, the valence by heavier competitors interaction, telomere length at d4, developmental telomere attrition (*D*) and the valence by developmental telomere attrition interaction. Since the valence by telomere attrition interaction was not significant, we excluded this interaction from the final model for which we present parameter estimates below. Speed significantly predicted latency to probe with birds that had faster mean speeds probing POS and NEG also probing ambiguous stimuli faster (GLMM: B ± se = 0.07 ± 0.01, X^2^(1) = 31.53, p < 0.001). Valence also significantly predicted latency to probe with birds probing stimuli more similar to POS faster (GLMM: B ± se = -0.70 ± 0.08, X^2^(1) = 89.82, p < 0.001). Number of heavier competitors was not significant, but the interaction between valence and number of heavier competitors reported above remained significant (GLMM: B ± se = 0.08 ± 0.03, X^2^(1) = 7.94, p = 0.005). Telomere length at d4 was marginally non-significant, with birds with shorter telomeres at d4 tending to probe faster (GLMM: B ± se = 0.24 ± 0.12, X^2^(1) = 3.23, p = 0.072). Telomere attrition, *D*, significantly predicted latency to probe, with birds that had suffered greater developmental telomere attrition probing ambiguous stimuli faster (GLMM: B ± se = 0.41 ± 0.20, X^2^(1) = 4.02, p = 0.045; Fig 2). The fit of the model including developmental telomere attrition was better than the model containing just number of heavier competitors as a predictor when the two models were run on the same restricted data set (ΔAIC_c_ = -1.88) arguing for the retention of developmental telomere attrition in our final model of the birds’ behaviour towards ambiguous stimuli. Note that the effect of developmental telomere attrition reported here is qualitatively the same if the model is run without number of heavier competitors included (statistics not shown). We can thus conclude that number of heavier competitors and developmental telomere attrition appear to have independent and opposite effects on the birds’ relative latencies to probe ambiguous stimuli.

#### Effects of genetic family

**Fig 3:**
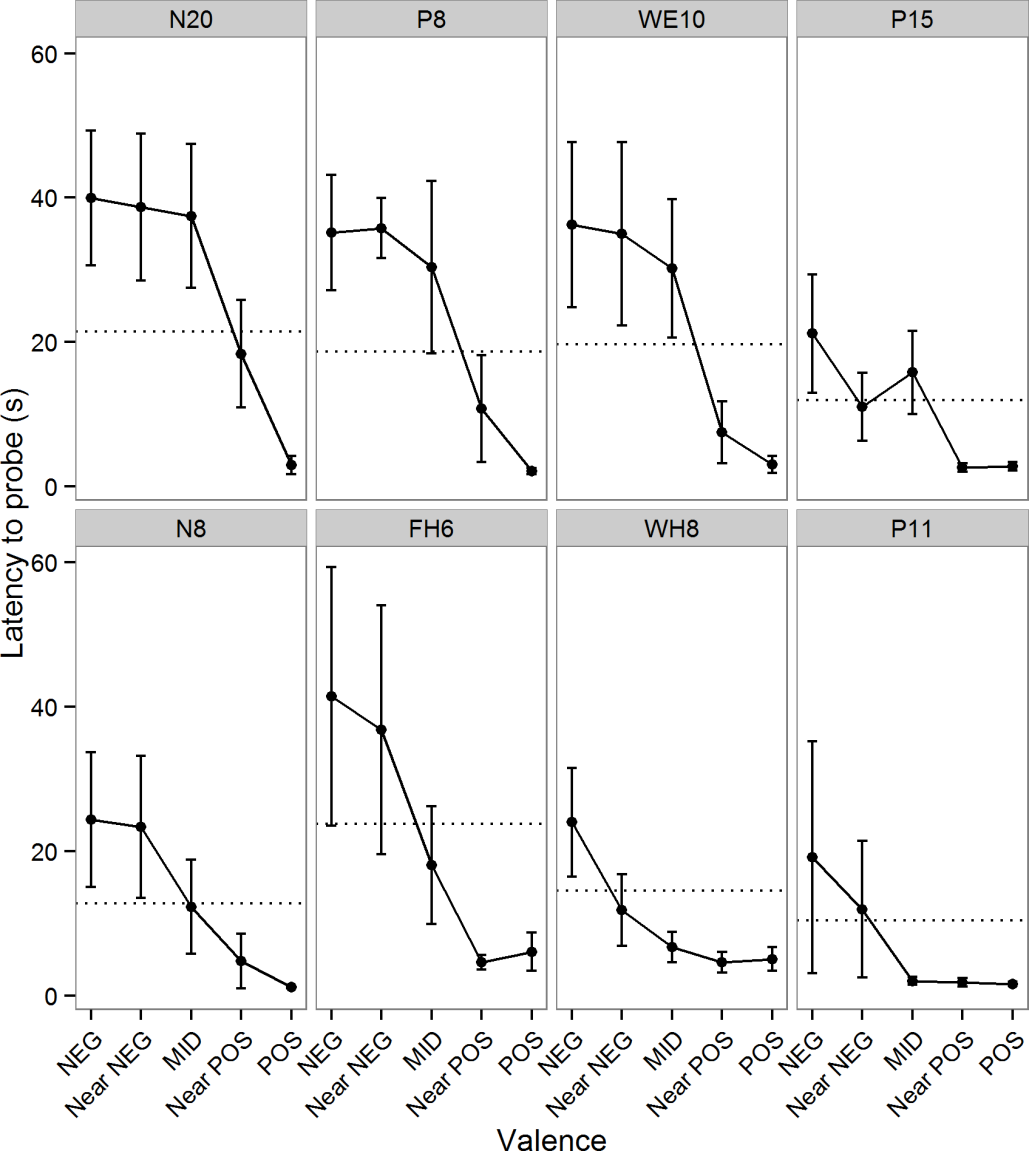
Effects of genetic family on judgment bias. Latency to probe as a function of stimulus valence for each of the 8 genetic families. Families FH6 and P11 contained 3 birds and the other families contained 4 birds. Data are mean ± 1 s.e. latency to probe in the judgment bias test trials. The dotted lines show the mean speed for each family (the mean of the mean latencies to probe POS and NEG). The top row of families were ‘pessimists’, meaning that their mean latencies to probe ambiguous stimuli were predominantly greater than the mean speed and more similar to NEG, whereas the bottom row were ‘optimists’, meaning that their mean latencies to probe ambiguous stimuli were predominantly less than the mean speed and more similar to POS.

In all preceding analyses we included genetic family as a random effect because we assumed that birds from the same family were likely to be more similar to each other than birds chosen at random, but this assumption was not tested explicitly. Fig 3 shows the cognitive bias data split by family. To test whether mean speed differed between families, we fitted a model with speed as the dependent variable and family as a random effect. Family explained none of the variance in speed, and there was no significant change in deviance when family was dropped from the model (GLMM: X^2^(1) = 0.00, p = 1.000). To test whether responses to ambiguous stimuli in particular differed between families, we fitted a model with latency to probe (logged) as the dependent variable, speed and valence (2–4) as fixed predictors and family and bird as nested random effects. Genetic family explained 6.4% of the variance and there was a significant change in deviance when family was dropped from the model (Χ^2^(1) = 4.78, p = 0.029), suggesting a significant effect of genetic family.

### Comparison of the current data set with other data from the same birds

Following the experiment reported in the current paper, we went on to collect further behavioural data from the same birds that are reported elsewhere. Of specific relevance to the current results, we found that birds with greater developmental telomere attrition were also more impulsive on a delay discounting task, showing a stronger preference for smaller but sooner food rewards [25]. Given our finding in the current paper that greater developmental telomere attrition appears to be associated with higher levels of optimism about potential reward, we sought to explore the nature of the associations between optimism/pessimism as measured by the current cognitive bias task and the impulsivity measured in our previously published experiment. We also included in this analysis the birds’ mean speed to probe POS and NEG stimuli, because this is yet another behavioural variable that appears to reflect the motivation of the birds to access food rewards.

In order to explore the relationships between pessimism, mean speed and impulsivity we needed a single number that provides an index of pessimism for each bird. To this end, we computed each bird’s mean latency to probe all three intermediate stimuli and expressed this as a proportion of the difference between its mean latencies to probe the POS and NEG stimuli: pessimism index = (mean intermediate latency − mean POS latency)/(mean NEG latency − mean POS latency) [40]. This pessimism index is equal to zero if a bird responds to the intermediate stimuli at the same speed as to the POS stimuli, and to one if it responds to the intermediate stimuli at the same speed as to the NEG stimuli; thus higher values indicate greater pessimism about reward.

Table 4 shows the correlation matrix between the pessimism index, mean speed and impulsivity for the subset of birds for which we had all three behavioural variables (n = 27). Impulsivity is measured by the value, *k*, reported in Bateson et al. [25], where higher values of *k* indicate more impulsive birds. Although none of the individual correlations is significant, the correlation coefficients show that birds that are less pessimistic (i.e. relatively faster to probe ambiguous stimuli) are also faster to probe POS and NEG stimuli and more impulsive on a delay discounting task, suggestive of a single trait describing motivation to access food reward. To test this idea, we conducted a PCA on the three variables without rotation. The Kaiser-Meyer-Olkin (KMO) measure verified the sampling adequacy for the analysis (KMO = 0.55 which is deemed adequate for PCA to yield distinct and reliable factors by Field [44]), and all KMO values for individual variables were also > 0.5 (again, above the acceptable limit for PCA [44]). The PCA extracted one factor with an eigenvalue > 1 that explained 42% of the variance. This single factor was retained in the analysis. Factor loadings were: pessimism = 0.69, mean speed = 0.56 and impulsivity = -0.69. Thus, positive values of the factor identify animals that were pessimistic, slow to probe trained stimuli and not impulsive about immediate reward, whereas negative values identify animals that were optimistic, fast to probe trained stimuli and impulsive about immediate reward. Based on these characteristics, we called the factor the ‘hunger factor’, since negative values seem to capture the behaviour of animals that are acutely hungry.

**Table 4:** Correlation matrix for pessimism index, mean speed and impulsivity.

	Pessimism index	Mean speed
**Mean speed**	r = 0.11	
	n = 27	
	P = 0.580	
**Impulsivity**	r = -0.18	r = -0.11
	n = 27	n = 27
	P = 0.374	P = 0.590

Notes. The correlation matrix was produced for the subset of birds for which all three behavioural measures were available (n = 27). Each cell contains: the Pearson product-moment correlation coefficient (r), the number of birds on which the test is based (n) and the P-value for the correlation (P).

**Fig 4:**
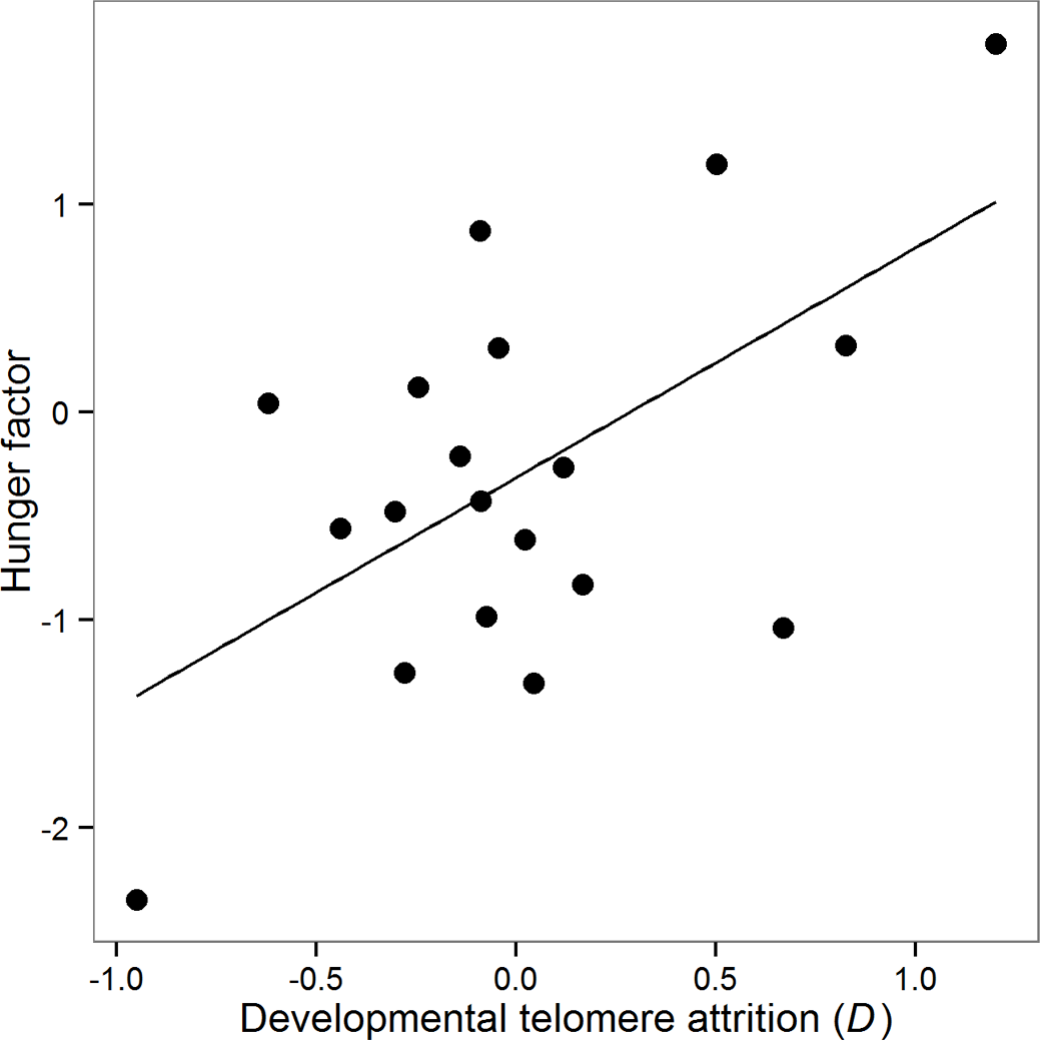
Effects of developmental telomere attrition on the ‘hunger factor’. Data points are the scores extracted by the PCA (see text for details) for each of the birds for which we also had telomere attrition data (n = 19). Negative values of the hunger factor indicate birds with a ‘hungrier’ cognitive phenotype, whereas positive values indicate a more sated cognitive phenotype. The solid line shows the predicted regression line derived from a simple regression.

To explore whether the hunger factor is predicted by developmental telomere attrition we fitted a model with the hunger factor (logged) as the dependent variable and telomere length at d4 and developmental telomere attrition (*D*) as fixed predictors. The data used for this analysis were from the subset of the birds used in the PCA for which we additionally had telomere length data (n = 19). Telomere length at d4 was not significant (GLMM: B ± se = -0.11 ± 0.12, X^2^(1) = 1.00, p = 0.318), whereas telomere attrition, *D*, significantly predicted the hunger factor, with birds that had suffered greater developmental telomere attrition behaving as if hungrier (GLMM: B ± se = 0.45 ± 0.17, X^2^(1) = 6.42, p = 0.011; Fig 4).

## Discussion

We set out to explore whether starlings’ developmental experience when they were chicks had an impact on their mood as juveniles. We measured mood using a cognitive bias task that was designed to measure expectations of punishment and reward when birds were presented with ambiguous stimuli intermediate between two stimuli previously associated with punishment and reward.

Before discussing the results obtained from the cognitive bias task, some methodological issues relating to the design and analysis of the task need to be considered. The task we used was based on a task originally developed for starlings by Bateson and Matheson [15]. The rationale underlying this go/no-go task was that birds would learn to probe the stimulus associated with reward (palatable mealworm) and to avoid the stimulus associated with punishment (a toxic, quinine-injected worm). In our previous use of this task with adult birds, the starlings rapidly learned not to flip the lids associated with the toxic worm, as would be expected if quinine is a punisher. However, in the current experiment the birds continued to probe the quinine-associated stimulus and, in many cases, ate the quinine-injected worm, even after extensive training. The fact that their latencies to probe quinine-associated stimuli were significantly greater than their latencies to probe the stimuli associated with palatable worms rules out the possibility that they were unable to learn the discrimination, and instead suggests that the quinine was not a punisher for the birds, perhaps because the toxic worms additionally contained valuable nutrients such as protein and fat. This conclusion fits with previous results showing that both acutely hungry starlings [45,46] and starlings from large broods with a ‘memory of hunger’ [42] will strategically choose to consume quinine-injected worms. The current study took place during the period when the birds were growing their adult plumage, perhaps explaining an increased protein requirement that was satisfied by the mealworms. The fact that our cognitive bias task did not contain a punisher, but instead, two rewards of differing value has implications for what the task measures. If the task contained no punisher, then it could not measure expectation of punishment, and by extension, it could not assess anxiety-like mood. We therefore suggest that the results from the current experiment are better interpreted as relating to expectation of reward only, and are hence most relevant to individual differences in depression-like moods. It is worth noting that many published cognitive bias tasks do not contain punishers [47,40,48], therefore our task is not unique in this respect, and the absence of a punisher in no way devalues the information provided by the task about expectation of reward.

A second methodological issue that requires discussion concerns the best method for analysing the data produced by cognitive bias experiments of this type. Gygax [29] has recently produced a set of recommendations, and argues that it is “conceptually flawed” to analyse responses to ambiguous stimuli separately from responses to the trained stimuli, because the trained stimuli are needed to anchor meaningful estimates of slope across the ambiguous stimuli. Gygax suggests that the fitted curve should be sigmoidal and that statistical techniques should account for censored data. We disagree with his conclusions for a number of theoretical and pragmatic reasons. First, our hypotheses concern individual differences in responses to the ambiguous stimuli, not the trained stimuli. Hence the analysis should focus on the ambiguous trials. Second, the trials with ambiguous stimuli are different, because they are given at low frequency, and are never reinforced, unlike the trials with the POS and NEG stimuli for which the birds have been extensively trained. This methodological difference argues against pooling data from the two trial types in any analysis. Third, the censoring of latency data due to probe latencies exceeding the maximum trial length is greatly reduced if only data from ambiguous trials are analysed, removing the need for analyses techniques designed for censored data [29,49]. Fourth, fitting sigmoidal curves to latency data, which is necessary to detect hypothesised effects if all stimuli are included, is statistically complex (as acknowledged by Gygax [29]). However, this complexity is removed if only the ambiguous trials are analysed, because differences in either the slope or intercept of straight lines can adequately capture treatment differences in cognitive bias. Finally, if there are treatment effects on how subjects respond to the trained stimuli, as was the case in the current data set, it makes sense to control for these effects by using the data from the trained stimuli as a covariate in the analysis of the ambiguous stimuli. For these reasons, we chose to analyse the birds’ responses to the ambiguous stimuli separately from their responses to the trained stimuli in the current experiment (for a similar approach see [40]).

We predicted that birds who had more competitors in the nest as chicks, and specifically more heavier competitors, would display cognitive changes indicative of more negative affective states. Specifically, we predicted that these latter birds would show evidence of a lower expectation of reward when tested with ambiguous stimuli. The results showed two distinct effects of developmental competition that we discuss in the following two paragraphs.

The first result, which we did not anticipate, was that birds that had experienced higher competition as chicks were faster to probe the unambiguous trained stimuli, a difference particularly evident for the NEG stimulus (Fig 1A). This result fits with other findings from the same cohort of birds showing that the developmentally-disadvantaged individuals were more prepared to eat unpalatable prey, similar to acutely hungry animals [42]. It also fits with findings from rats showing that developmentally stressed animals made faster choices on a cognitive bias task [31], and more generally with human epidemiological findings linking early-life adversity with the development of impulsive behaviour patterns [50].

The effect of number of competitors on latency to probe unambiguous stimuli described above, complicated the analysis of the cognitive bias data because it obscured differences in the shape of the generalisation gradients obtained for the ambiguous stimuli. To explore how developmental competition affected the birds’ responses to the ambiguous stimuli, we controlled statistically for the difference in speed by including the mean latency to probe the trained stimuli as a covariate in our models. Our second result relating the effects of developmental competition was that, as predicted, the chicks with more, competitors, and specifically more heavier competitors, showed relatively greater latencies to probe the ambiguous stimulus most similar to the stimulus associated with reward (Fig 1B and 1D), suggestive of a relatively lower expectation of reward. Therefore, as predicted, birds that had been disadvantaged in early food competition, and had as a result experienced a reduced probability of reinforcement during development, showed evidence of reduced expectation of reward as independent juveniles. This result supports our hypothesis that developmental experience of probability of reward could shape adult reward response thresholds [4].

We also hypothesised that developmental telomere attrition, a putative marker of the cumulative effects of developmental stress and measure of somatic ‘state’, might affect mood-related cognition. There is growing evidence from human epidemiological studies showing an association between shorter telomeres, pessimistic cognitive styles, and affective disorders including generalised anxiety disorder and major depression [51,52]. These data led us to predict that birds with greater telomere attrition would have a lower expectation of reward in the cognitive bias task. However, whilst we found an effect of telomere attrition on birds’ responses to ambiguous stimuli, this was in the opposite direction to that predicted: birds with greater developmental telomere attrition between d4 and d55 had higher expectations of reward. This effect was independent of the previously described effect of number of heavier competitors. This result, linking greater telomere attrition to a higher expectation of reward, potentially fits with a later data set collected from the same cohort of birds in which we showed that developmental telomere attrition predicted greater impulsivity measured using a time preference task [25]. We therefore have two pieces of evidence from these birds linking greater developmental telomere attrition with altered adult decision making. To explore further the relationships between telomere attrition, optimistic cognitive biases and impulsivity we conducted a PCA on two ostensibly independent variables from the current experiment, mean speed to probe trained stimuli and the pessimism index, and *k*, our previously published measure of impulsivity from the same birds [25]. This PCA yielded a single ‘hunger factor’ that was itself strongly predicted by developmental telomere attrition, with birds that had experienced greater telomere attrition behaving as if they were hungrier (Fig 4). This result adds strength to our conclusion that poor somatic state, as measured *via* greater developmental telomere attrition, is associated with a cognitive phenotype characteristic of acutely hungry animals. Optimality models developed within behavioural ecology have long since predicted that animals should behave differently depending on their state (for a review see [53]). However, exactly what ‘state’ is and how we should measure it has been less clear. Telomere attrition is emerging as a plausible cellular biomarker of somatic state, and it is therefore encouraging that it appears to predict behavioural decisions in line with the predictions of some optimality models (see also [25]). Further work will be needed to clarify how well telomere attrition holds up as a predictor of individual differences in decision making.

Thus, the picture arising from the cognitive bias data is complex. On the one hand, in line with our predictions, we have evidence linking past developmental stress with increased ‘pessimism’ about reward. Birds that had experienced greater competition in the nest were relatively slower to probe ambiguous stimuli most similar to the trained stimulus associated with reward. On the other hand, contrary to our predictions, we have evidence linking a biomarker of poor somatic state with increased ‘optimism’ about reward. Birds with greater telomere attrition were faster to probe ambiguous stimuli. This latter finding in particular leads us to question whether the assumed association between expectation of reward and the valence of affective state made in the cognitive bias literature is always valid [8,27]. Rather than seeing birds that probe ambiguous stimuli as if they were positive stimuli as necessarily ‘optimistic’, we could view them as desperate for reward, or as risk-prone. Theoretical models show that it can be adaptive for animals in a poor current state to be risk-prone with regard to gaining rewards [54,55]. The potential link between risk-prone behaviour and ‘optimism’ as measured by cognitive bias tasks clearly needs further exploration.

In summary, we have shown that one measure of early-life adversity—having a larger number of competitors heavier than oneself—leads to relative ‘pessimism’, and another—telomere attrition—is associated with relative ‘optimism’ about reward possibly better described as desperation or risk-proneness. We set out by identifying two factors that should theoretically influence response thresholds for ambiguous rewards and hence the ‘pessimism’ of decisions, namely, the estimated probability of reward and the vulnerability of the individual. We hypothesised that both of these factors could plausibly be influenced by early-life adversity. The results described above could be interpreted as suggesting that early disadvantage led birds to estimate a reduced probability of reward, whereas poor somatic state led them to value food reward more highly, hence explaining the opposing effects of the two variables on cognitive bias.

Our data show evidence of causal links between developmental experience, somatic state, and cognitive bias, confirming that development is important in determining adult moods (as currently measured). However, the presence of these associations says nothing about whether these effects are pathological or adaptive. The speed of associative learning is often used as a sensitive measure of cognitive ability and could be used to assess whether our developmental manipulation produced any evidence of general cognitive impairment in the birds [56–58]. The training required for the judgment bias task provides two potential measures of the speed of associative learning: first, the number of trials taken to acquire the operant lid flipping, and second, the number of trials taken to acquire the discrimination between stimuli associated with palatable and toxic worms. We found no evidence for an effect of developmental experience on either of these measures. These results agree with other data sets on the same cohort of birds [25,42], neither of which have found any evidence for an effect of early competition on learning performance. Given the lack of evidence for impairments in learning ability, we conclude that the cognitive differences that we found in cognitive bias are unlikely to be symptomatic of general cognitive impairment, but are perhaps more likely to be adaptive responses to subtle changes in experience or somatic state.

As a final point, the design of our brood size manipulation with quartets of siblings additionally allowed us to explore whether there were family differences in behaviour on the cognitive bias task. We found no effects of genetic family on overall probing speed. However, we did find significant effects of genetic family on cognitive biases, with some families having a more ‘optimistic’ and some a more ‘pessimistic’ cognitive style (Fig 3). These differences are independent of the treatment and telomere effects discussed previously. Whether these differences are genetic in origin is impossible to determine from the current data set, but their cause must predate the cross-fostering of chicks that took place on day 3.

## Supporting Information

S1 File Data by bird. (CSV)

S2 File Cognitive bias test data by trial. (CSV)

S3 File Definitions of variables included in S1 File and S2 File. (TXT)
